# Correlation among Metabolic Changes in Tea Plant *Camellia sinensis* (L.) Shoots, Green Tea Quality and the Application of Cow Manure to Tea Plantation Soils

**DOI:** 10.3390/molecules26206180

**Published:** 2021-10-13

**Authors:** Litao Sun, Kai Fan, Linlin Wang, Dexin Ma, Yu Wang, Xiaojun Kong, Hongyan Li, Yonglin Ren, Zhaotang Ding

**Affiliations:** 1 Tea Research Institute, Qingdao Agricultural University, Qingdao 266109, China; slttea@163.com (L.S.); fankaitea@163.com (K.F.); LinlinWangtea@163.com (L.W.); wangyutea@163.com (Y.W.); 2 College of Science, Health, Engineering and Education, Murdoch University, 90 South Street, Perth, WA 6150, Australia; 3 College of Communication, Qingdao Agricultural University, Qingdao 266109, China; madexin@163.com; 4 Rizhao Tea Technology Promotion Center, Rizhao 276826, China; kxj006@163.com; 5 Haiyang Fruit Industry Technology Promotion Station, Haiyang 265100, China; hygy059@163.com

**Keywords:** *Camellia sinensis* (L.), fertilization, metabolite, cow manure, tea quality

## Abstract

Traditionally, the supplement of organic manure in tea plantations has been a common approach to improving soil fertility and promoting terroir compounds, as manifested by the coordinated increase in yield and quality for the resulting teas. However, information regarding the effect of organic manure in the metabolome of tea plants is still inadequate. The metabolite profiles of tea shoots applied with cow manure, urea or no fertilizer were studied using gas chromatography–mass spectrometry (GC–MS). In total, 73 metabolites were detected, and the modulated metabolites included mainly amino acids, organic acids and fatty acids. In particular, glutamine, quinic acid and proline accumulated more in tea shoots in soils treated with cow manure, but octadecanoic acid, hexadecanoic acid and eicosanoic acid were drastically reduced. Pearson correlation analysis indicated that organic acids and amino acids in tea shoots were the two major metabolite groups among the three treatments. The analysis of metabolic pathways demonstrated that the cow manure treatment significantly changed the enrichment of pathways related to amino acids, sugars and fatty acids. Sensory evaluation showed that the quality of green teas was higher when the plants used to make the tea were grown in soil treated with cow manure rather than urea during spring and late summer. The results indicated that the application of cow manure in soils changed the metabolic characteristics of tea shoots and improved the qualities of the resulting teas.

## 1. Introduction

The tea plant, *Camellia sinensis* (L.) O. Kuntze, is an evergreen leafy plant that is used in the manufacturing of beverages. The production and consumption of tea worldwide has continually increased over decades. China is a major tea producer and tea exporter, with a total export value of 13 billion USD and production accounting for approximately 45% of the global total output in 2019 [1]. Green tea, accounting for the largest share of the tea industry, is widely appreciated for its health properties, elegant flavor and pleasant aroma [2].

Tea quality depends on the metabolites produced by various metabolic pathways [3]. For example, polyphenolic compounds contribute bitterness and astringency [4]; amino acids are related to the umami of tea infusions [5]; the presence of sugar is crucial for the synthesis of catechin and soluble solids [6] and fatty acids are well-known precursors of aroma compounds such as hexanal, (E)-2-hexanol and methyl jasmonate [7], which are produced in various metabolic pathways. Considering the primary metabolism, partial compounds in tea leaves were involved in both nitrogen (N) and carbon metabolism processes [8]. For instance, the accumulation of amino acids in tea leaves has been considered as the result of the regulation of nitrogen metabolism. Secondary compounds in tea leaves tends to be more abundant, and multiple secondary metabolisms, such as the phenylpropanoid pathway and the flavonoid pathway, were found to be involved in the synthesis of these compounds [9].

It is well known that the levels of significant compounds in tea leaves are influenced by many factors, including field management and time of season. The supplement of organic fertilizers in tea plantations is a common approach to improve yield and quality of teas. However, a growing emphasis is being placed on the positive aspects of the application of organic fertilizers as large amounts of the remaining chemical fertilizers leak into the environment causing water pollution and soil acidification [10,11]. Compared to chemical fertilizers, organic fertilizers have a harmonious combination of nutrients [12,13], modifying the physical, chemical and biological properties of soils [7] and increasing the fertilizer utilization efficiency [14]. Organic fertilizers also play an important role in improving the yield and quality of teas. Previous studies indicated that substituting 25% of nitrogen fertilizers with organic fertilizers (pig manure) was recommended to achieve the highest yield with the lowest labor cost (for the manual application of organic fertilizers) [15]. Proper replacement with organic fertilizers (rape seed cake, 50%) can effectively promote the yield and quality of teas [16]. However, information regarding the effect of organic fertilizers on the metabolism of tea plants is still inadequate.

In our study, we conducted a field experiment to analyze the modulated metabolites in tea shoots over three seasons (spring and early and late summer) with no fertilizer as well as with urea and cow manure soil treatments. The objectives were (1) to determine the changes of metabolites in tea shoots during the growing seasons, (2) to determine the metabolic pathways in tea shoots in soils treated with cow manure, and (3) to evaluate the impact of cow manure on the quality of teas.

## 2. Results

### 2.1. Analysis of Metabolic Profiling in Tea Shoots under Different Treatments

**Figure 1:**
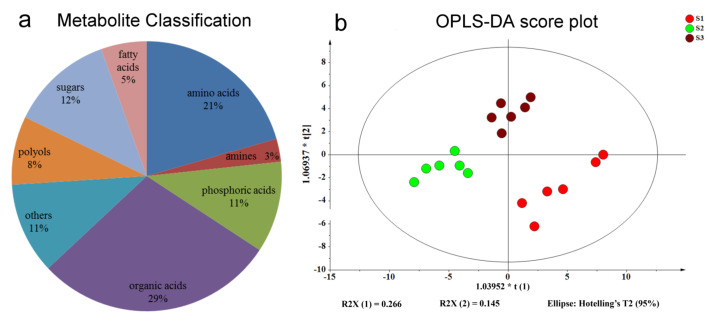
Pie chart of metabolite classification (**a**) and OPLS-DA score plots for metabolic profiling analysis (**b**). The score of variation explained by each principal component is indicated on the axes. Each point corresponds to a plant sample, and different colors indicate the different treatments. R2X-1 and R2X-2 are the degrees of explanation on the first and second principal component axes, respectively; t-1 and t-2 are the first and second principal component axes, respectively. Ellipses represent the 95% confidence regions for each subclass of observations, assuming normal distributions.

To study the influences of different fertilizers on the metabolome of tea shoots, the metabolite profile was identified using GC–MS analysis. In total, 134 putative metabolites were observed in tea shoots. Among them, 73 compounds could be identified using the National Institute of Standards and Technology (NIST) and Wiley libraries (Table S1), including organic acids 29%, amino acids 21%, sugars 12%, phosphoric acids 11%, polyols 8%, fatty acids 5%, amines 3% and others 11% (Figure 1a).

In order to visualize the data set, orthogonal-partial least squares-discriminant analysis (OPLS-DA) was preformed, and the results demonstrated that S1, S2 and S3 had extensive differences in metabolite profiles (Figure 1b). Comparing the three treatments, the major metabolites that contributed to the first principal component (PC1) were ribonic acid and caffeine. The contribution of metabolites to the second principal component (PC2) was dominated by octadecanoic acid, hexadecanoic acid, salicylic acid, mannose, glucose and proline (Figure S1).

### 2.2. Analysis of Metabolite Correlations in Tea Shoots under Different Treatments

To investigate the metabolite–metabolite correlation in tea shoots, we calculated the data for different metabolites in S1, S2 and S3 using Pearson correlation coefficient. The results showed some marked associations among the same or different metabolites (Figure S2). In total, 498 significant correlations were found, including 349 positive and 149 negative correlations.

**Figure 2:**
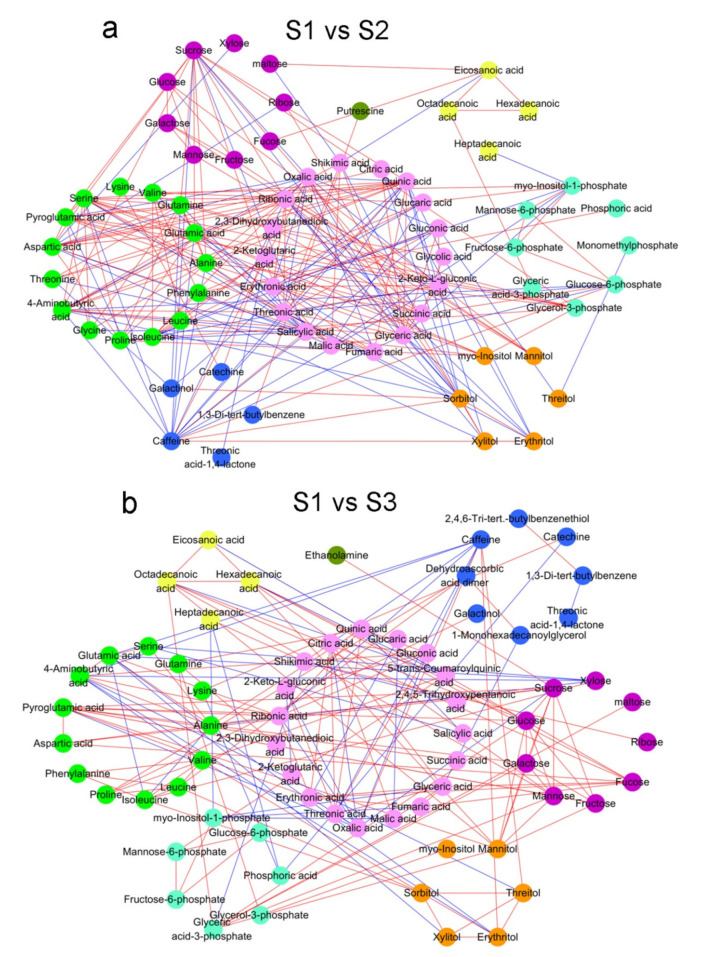

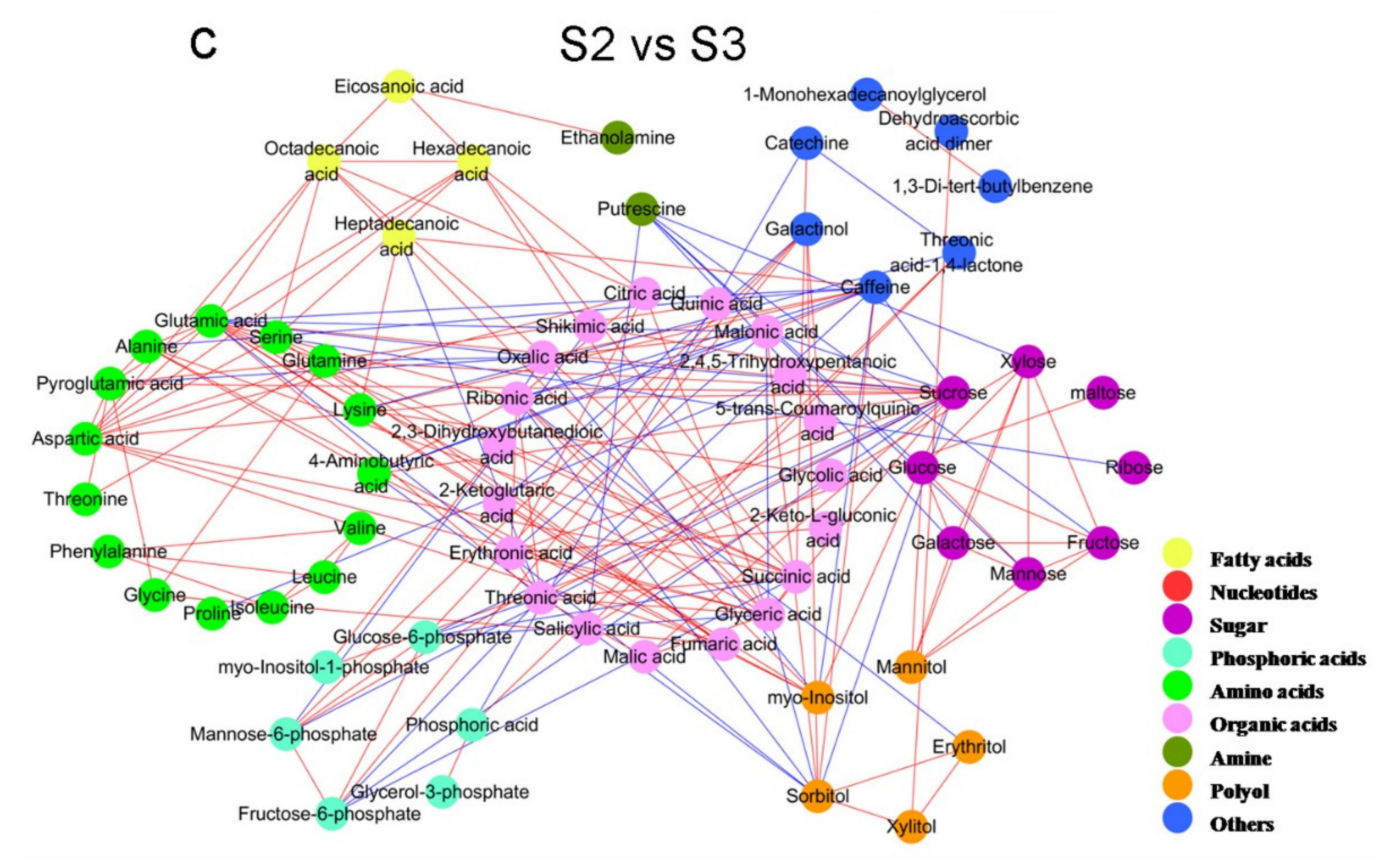
Network map of correlation analysis among difference metabolites: (**a**) S1 vs. S2; (**b**) S1 vs. S3; (**c**) S2 vs. S3. Metabolites are represented by circles. The same color indicates metabolites in the same metabolic function group. Correlations are shown by connected lines. Red lines are positive correlations, and blue lines are negative correlations.

Among the correlations of different metabolites, organic acids and amino acids were the two major metabolite groups (Figure 2). Compared to S1, 19 organic acids in S3 involved in 42 significant correlations, including 22 positive correlations and 20 negative correlations (Figure 2b). Thirteen amino acids were involved in 49 significant correlations, including 38 positive correlations and 11 negative correlations. Additionally, 28 positive and 3 negative correlations were related to sugars. To the lesser groups, fatty acids in S3 were involved in seven significant correlations, including three positive and four negative correlations. Compared to S2, the numbers of positive correlations in S3 related to organic acids, amino acids, sugars and fatty acids were 49, 42, 25 and 18, respectively, and the numbers of negative correlations in S3 related to organic acids, amino acids, sugars and fatty acids were 25, 7, 11 and 1, respectively (Figure 2c).

### 2.3. Analysis of Difference Metabolites in Tea Shoots under Different Treatments

To further identify the differences among metabolites in S1, S2 and S3, metabolites were chosen by fold change (FC ≥ 1.5 or FC ≤ 0.667) with significant difference (*p* < 0.05). Here, some modulated metabolites were found through comparisons of each group after screening; these metabolites included mainly amino acids, organic acids and fatty acids (Table 1).

Compared to those in S1, most of the identified metabolites in S3 had more accumulations, including glutamine, quinic acid and proline; however, the levels of octadecanoic acid, hexadecanoic acid, salicylic acid and eicosanoic acid decreased significantly. Compared to S2, the contents of amino acids, fatty acids, sugars, polyols and phosphoric acids in S3 Were noticeably decreased, especially the levels of glutamine, sucrose, octadecanoic acid, pyroglutamic acid, alanine and hexadecanoic acid.

### 2.4. Analysis of Metabolic Pathway in Tea Shoots under Different Treatments

**Figure 3:**
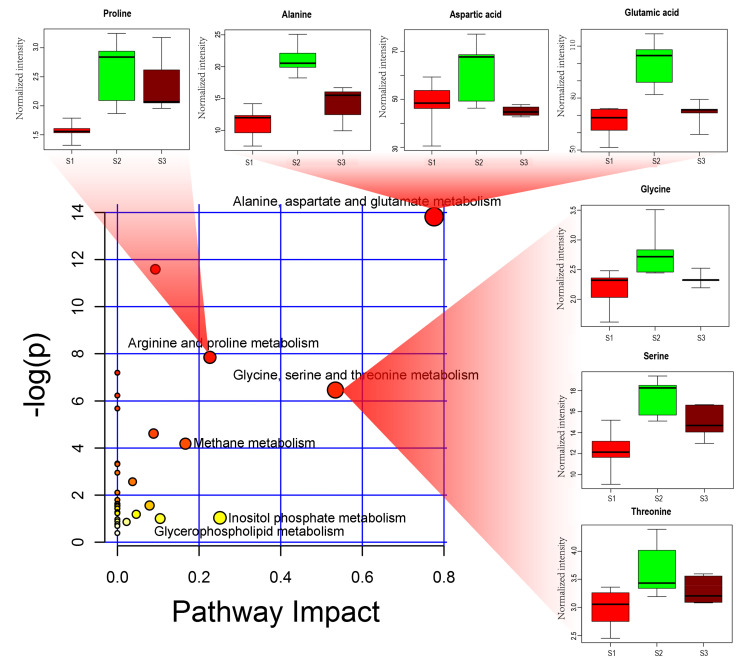
Pathway analysis shows the impact of different metabolisms and the change of compounds in amino acid metabolism. The y-axis boxplots (–) indicate the median values, the top/bottom ranges of the boxes indicate the upper/lower quartiles, and the top/bottom whiskers indicate the maximum/minimum distribution of the data. The boxes across the x-axis from left to right indicate the tea shoots of S1 (red), S2 (green) and S3 (brown), respectively (for interpretation of the references to color in this figure legend, the reader is referred to the web version of this article).

According to analysis of the Kyoto Encyclopedia of Genes and Genomes (KEGG) enrichment, six significantly enriched pathways (Impact > 0.1) were identified, including alanine, aspartate and glutamate metabolism; glycine, serine and threonine metabolism; inositol phosphate metabolism; arginine and proline metabolism; methane metabolism and glycerophospholipid metabolism (Figure 3).

**Figure 4:**
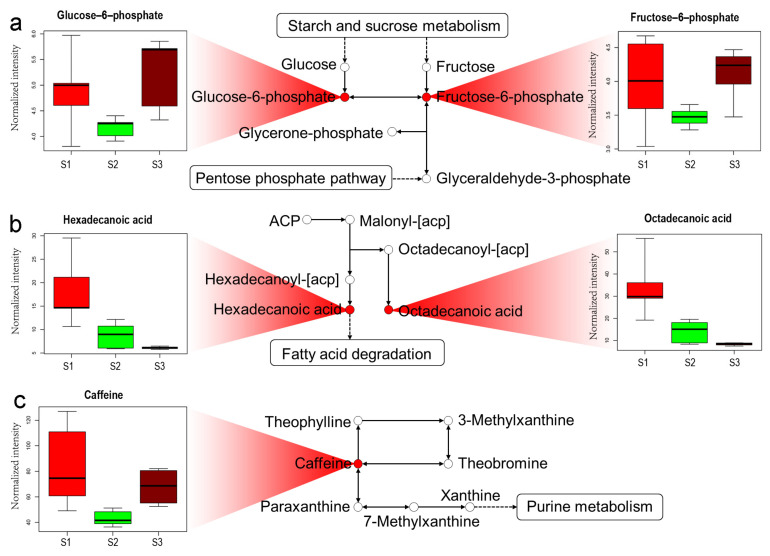
The metabolic pathways were affected mainly by different fertilizations: (**a**) glycolysis/gluconeogenesis; (**b**) fatty acid biosynthesis; (**c**) caffeine metabolism. The y-axis boxplots (–) indicate the median values, the top/bottom ranges of the boxes indicate the upper/lower quartiles, and the top/bottom whiskers indicate the maximum/minimum distribution of the data.

Amino acid metabolism was mainly enriched across the three samples (S1, S2 and S3). For example, compared to S1, the relative quantities of alanine, glutamic acid, glycine, serine, threonine and proline in S3 increased by about 21%, 8%, 8%, 18%, 10% and 34%, respectively. Unexpectedly, compared to S2, the contents of these amino acids were 33%, 9%, 16%, 14%, 10% and 29% lower in S3 (Table S2), respectively. Regarding glycolysis/gluconeogenesis, the contents of several carbohydrates (such as glucose-6-phosphate and fructose-6-phosphate) increased in S3 compared to S1 and S2 (Figure 4a). Regarding biosynthesis of fatty acids, the relative quantities of octadecanoic acid and hexadecanoic acid were 75% and 66%, which were lower in S3 than that in S1 (Figure 4b). Regarding caffeine metabolism, the content of caffeine also decreased in S3 (Figure 4c).

### 2.5. Sensory Evaluation of Green Teas under Different Treatments

To determine the effect of different treatments on tea qualities, we evaluated the sensory quality of green teas made from plants harvested during three seasons, namely, spring, early summer and late summer (Table 2). In spring, the highest score for sensory evaluation was observed in S3 on all parameters, including appearance (87.67), aroma (90.33), infusion color (88.33), infusion taste (89.00) and infused leaf (88.00), indicating better quality in S3 than S2 and S1. In early summer, however, the lowest score was obtained in S3, and the highest score was obtained in S2. In late summer, it was noteworthy that the scores of S3 for appearance (92.00), aroma (90.33), infusion color (90.00), infusion taste (90.00) and infused leaf (88.33) were distinctly higher than those of other samples.

## 3. Discussion

In this study, soils treated with cow manure led to significant changes of metabolites in tea shoots. These metabolites were enriched in several metabolic pathways, including pathways for amino acids, sugars and fatty acids (Figure 3 and Figure 4). The results showed that the application of cow manure in soils in tea plantations was favorable in improving the quality of teas (Table 2).

### 3.1. The Application of Cow Manure Affects the Amino Acid Metabolism in Tea Shoots

Amino acids, the main metabolites of tea leaves, are among the most important determinants of tea quality. Changes in amino acids are influenced by many factors, such as climate, altitude and soil nutrition. Dai et al. [10] and Xu et al. [17] studied the differences among metabolites in green teas during spring, early and late summer. They found that teas harvested during spring generally contained higher levels of free amino acids, whereas teas harvested during early and late summer contained lower levels. Ding et al. [18] reported that the content of glutamate decreased, and the content of glutamine increased in tea plants treated with P-deficiency, while the content of glutamine decreased treated with P-excess. Chen et al. [8] found that shade management (dark treatment) significantly increased the content of free amino acids in tea leaves. However, there is little information about the effects of organic manure on the content of amino acids in tea leaves. In our study, the contents of some amino acids (alanine, glutamic acid, glycine, serine, threonine and proline) increased in tea shoots harvested from soils treated with cow manure and urea, whereas the content of amino acids increased slowly in tea shoots harvested from soils treated with cow manure (Figure 3 and Table S2). This may influence the quality of green teas, because amino acids are the primary factor in the umami of tea infusions [19,20]. The sensory evaluation indicated that the scores of teas harvested during spring and late summer from soils treated with cow manure were higher than that of teas harvested from soils treated with urea. However, the scores of teas harvested in early summer were slightly lower in teas harvested from soils treated with cow manure (Table 2). Accordingly, in order to improve the umami of green teas for all harvest seasons, we suggest that when cow manure is applied to the soil of tea plantations, the proper amount of urea should be supplemented coordinately.

### 3.2. The Application of Cow Manure Affects the Sugar Metabolism in Tea Shoots

Sugars, as the primary compounds of photosynthesis, are the main source of organic carbons in plant growth. The levels of carbohydrates, especially those involved in cell wall composition in mature leaves (glucuronic acid, rhamnose and xylopyranose), decreased greatly from the early to the late periods during the spring tea season [3]. The abundant sugars transported from tea leaves to floral meristems provide energy for flower development [21]. Sugars are also considered as a determinant of tea quality; the increase in total soluble sugars improved the sweetness and mellow taste in tea [22]. Soluble sugars were found to be involved in the Maillard reaction, which forms many roasted flavor compounds and effectively improves the sensory quality of teas [23,24]. The influences of growth environment and seasonal harvest on carbohydrate partitioning had a significant effect on quality of bush tea [25]. In our study, the content of sucrose increased in tea shoots harvested from soil treated with cow manure and urea (Table 1). The carbohydrates related to glycolysis/gluconeogenesis, such as glucose-6-phosphate and fructose-6-phosphate, accumulated more in tea shoots harvested from soils treated with cow manure. However, the contents of these sugars decreased dramatically under urea treatment (Figure 4a). The sensory evaluation of green teas made from plants harvested from soils treated with cow manure demonstrated that the score for infusion taste was higher in spring and late summer, while the infusion taste under urea treatment was better only in early summer (Table 2). The results indicated that the application of cow manure was more beneficial to the accumulation of sugars in tea shoots.

### 3.3. The Application of Cow Manure Affects the Fatty Acid Metabolism in Tea Shoots

Fatty acids are crucial quality precursors in tea leaves and contribute greatly to the formation of aroma and flavor volatiles. Methyl jasmonate, an important fatty acid derivative, was a major contributor to the jasmine-like aroma of oolong teas [26]. The major aroma-active volatiles in black teas, such as (Z)-jasmone and dihydroactinidiolide, usually had sweet-like and fruit-like aromas [27]. Cis-jasmone and jasmine lactone usually had nutty-like or flower-like aromas in green teas [28]. Although the content of fatty acids and other lipids in tea leaves was low and made up only between 4 and 9% of the dry weight of fresh tea leaves [29,30], the metabolism of fatty acids was found to be important in the biogenesis of flavor [31,32]. Previous studies showed that fertilization could influence the formation of tea volatiles. Owuor et al. [33] demonstrated that high rates of nitrogenous fertilizer lowered the aroma quality of black tea partly because of the decrease in fatty acid content with increasing rates of nitrogenous fertilizer supplement. However, in green tea, high levels of these volatile flavor compounds are desired, as they improve the aroma of the product [16]. In our study, the levels of hexadecanoic acid, octadecanoic acid and eicosanoic acid decreased dramatically in tea shoots harvested in soils treated with cow manure and urea (Table 1). We realized that the above indication was not consistent with the results of sensory quality analysis of the resulting green teas. The aroma scores were the highest in teas harvested from soils treated with cow manure, except during early summer, but the aroma was not a significant characteristic under urea treatment (Table 2). The results showed that the application of organic manure can reduce the levels of fatty acids but enhance the level of aroma. Therefore, further research is needed regarding the composition of fatty acids and the correlation between fatty acids and aroma of teas.

According to the conjoint analysis between amino acid metabolism and fatty acid metabolism, an increase in amino acids was accompanied by a decline in fatty acids in tea leaves. Previous studies indicated that the assimilation of nitrogen into amino acids requires carbon skeletons, energy and reductants, which provided insight into the regulation of carbon flow as effected by nitrogen assimilation from a macro perspective [34,35]. However, related mechanisms in the balance between nitrogen, in the form of amino acids, and carbon, in the form of fatty acids, requires further investigation.

## 4. Materials and Methods

### 4.1. Field Trial

A trial field of tea plants located in Qingdao, North China Plain (36°19′ N, 120°23′ E, elevation 54.88 m), was selected to obtain samples for this study. The tea variety, *Camellia sinensis* (L.) cv. ‘Huangshanzhong’, had grown for more than 8 years. The field soil was classified as brown loamy soil with a pH value of 5.62. The major chemical parameters of the field soil were as follows: organic matter (OM, 8.17 g/kg), total nitrogen (TN, 1.18 g/kg), available nitrogen (AN, 68.49 mg/kg), available phosphorus (AP, 26.84 mg/kg) and available potassium (AK, 225.27 mg/kg). The experiment design included three field treatments: T1 (control experiment, unfertilized), T2 (urea, N: 46.67%) and T3 (cow manure, N: 1.50%). The nitrogen contents in cow manure and urea were determined using the routine methods of Brubaker [36]. The total nitrogen contents of T2 and T3 stayed the same (300 kg/ha) when the fertilizers were applied to the field by a one-off application.

### 4.2. Plant Sampling

Tea shoots (a bud and two expanding leaves) were collected from T1, T2 and T3, and the three corresponding plant samples were named S1, S2 and S3, respectively (each type with 3 replicates, 18 samples in total). The samples were picked by hand during three seasons, namely, spring (April), early summer (June) and late summer (August). These samples were processed into green teas for sensory evaluation according to the standardized procedure (GB/T23776-2009). All the green tea samples were blindly assessed by a tasting panel consisting of six officially certified tasters [3,37]. Briefly, five attributes were considered as main features for evaluating the quality of tea: appearance, color of the infusion, aroma, flavor and features of the infused leaves. Three grams of each manufactured tea sample were infused with 150 mL of freshly boiled water for four minutes. Scores were assigned for the shape of the dry tea (25%), the liquor color (10%), the aroma (25%), the flavor (30%) and the infused leaves (10%). Finally, the total scores of organoleptic qualities were calculated using the aforementioned weight values. Additionally, the tea shoots of S1, S2 and S3 collected in August were frozen quickly in liquid nitrogen and stored at −80 °C for metabolome analysis (each type with 6 replicates, 18 samples in total).

### 4.3. GC–MS Analysis

The metabolites of tea shoots were extracted by grinding 100 mg of the freeze-dried tissues in 5 mL of 80% methanol and then 60 µL of ribitol (0.2 mg/mL) added as an internal standard. The experimental procedure was described in detail by Jia et al. [21].

Raw gas GC–MS data was converted into NetCDF files by G1701 MSD ChemStation software and was subsequently processed by XCMS 3.5 (http://www.bioconductor.org, accessed on 30 August 2020). Peak detection and deconvolution were performed with the automated mass spectral deconvolution and identification system (AMIDS), and peak lists were compiled by the National Institute of Standards and Technology (NIST) and Wiley libraries. The resulting data matrix was subjected to multivariate analyses, and significant features were identified using MetaboAnalyst 4.0 (http://www.metaboanalyst.ca, accessed on 30 August 2020).

### 4.4. Pathway Analysis

Pathway analyses in MetPA were conducted through three routes. The procedure was described in detail by Xia et al. [38].

The identified metabolites were mapped onto biochemical pathways according to annotations in the Kyoto Encyclopedia of Genes and Genomes (KEGG; http://www.kegg.jp, accessed on 30 August 2020) and MetaboAnalyst 4.0. The pathway topology information and chemical compounds were parsed into graph models using the KEGG graph package [39]. Metabolic pathways were presented as a network of chemical compounds with metabolites as nodes and reactions as edges. The graph generation and manipulation were implemented using Cytoscape 3.5.1 software (http://www.cytoscape.org/, accessed on 30 August 2020).

### 4.5. Statistical Analysis

One-way analysis of variance (ANOVA) was used to examine the significant differences among the different treatments, with *p* < 0.05 being considered as statistically significant. An independent *t*-test was utilized to exclude variables that were significantly different (*p* < 0.05) among the different treatments (SPSS 18.0 software, Chicago, IL, USA). Subsequently, the Pearson correlation coefficient (PCC; r2 ≥ 0.49 and false discovery rate (FDR) ≤ 0.05) was used to analyze the correlations among different metabolites. The statistical test of significance was performed by cor.test function and was adjusted by *p* values in R software (version 3.2.1, Vienna, Austria). The heat map of correlation analysis (R/cor/cor.test) and boxplots (R/boxplot) were drawn by R software. The orthogonal-partial least squares-discriminant analysis (OPLS-DA) and principal component analysis (PCA) loading plot were available in the Soft Independent Modeling of Class Analogy (SIMCA)-P commercial software (Umetrics, Umea, Sweden) and R/ropls package [40].

## 5. Conclusions

In our study, we investigated the metabolic characteristics of tea shoots harvested in soils treated with cow manure. Some differences were found among metabolites by comparing three different field treatments (T1, unfertilized; T2, urea; T3, cow manure); these metabolites included mainly amino acids (glutamine, alanine and proline), organic acids (quinic acid and threonic acid) and fatty acids (hexadecanoic acid, octadecanoic acid and eicosanoic acid). The metabolic pathways related to amino acids, sugars and fatty acids changed significantly in tea shoots grown in soils treated with cow manure. The quality of teas harvested from soils treated with cow manure was better than that of other teas harvested during spring and late summer. We therefore recommend cow manure as an organic fertilizer for soils in tea plantations to improve the quality of teas.

## Figures and Tables

**Table 1:** Metabolite changes based on comparisons of different chemical groups.

Chemical Groups	Identified Chemicals	log2fc_S2/S1	log2fc_S3/S1	log2fc_S3/S2
Amino acids	alanine	**0.9265**	0.3433	**−0.5832**
	proline	**0.7331**	**0.6034**	−0.1297
	salicylic acid	**−0.6537**	**−0.7162**	−0.0625
	pyroglutamic acid	**0.7666**	0.1668	**−0.5998**
	4-aminobutyric acid	**0.6777**	0.2170	−0.4607
	glycine	0.3689	0.1127	−0.2562
	glutamic acid	**0.6056**	0.1152	−0.4905
	glutamine	**2.5405**	**1.1647**	**−1.3757**
	serine	**0.5055**	0.2923	−0.2132
	threonine	0.3040	0.1511	−0.1528
	aspartic acid	0.3761	−0.0801	−0.4562
Organic acids	succinic acid	0.4559	−0.0315	−0.4874
	quinic acid	**0.9348**	**0.7440**	−0.1908
	malonic acid	0.1325	0.3861	0.2536
	threonic acid	**0.7460**	0.2558	−0.4902
	glycolic acid	0.2467	0.3599	0.1132
Fatty acids	hexadecanoic acid	**−1.0449**	**−1.5632**	**−0.5183**
	octadecanoic acid	**−1.2808**	**−2.0232**	**−0.7425**
	eicosanoic acid	−0.4435	**−0.6362**	−0.1927
Sugars	Sucrose	**1.0156**	0.0591	**−0.9565**
Polyols	myo-inositol	**0.5233**	0.2252	−0.2982
Phosphoric acids	glycerol-3-phosphate	0.3133	0.3073	−0.0060
Others	Caffeine	**−0.9640**	−0.3172	**0.6468**

FC is fold changes described with the log2 transformed numbers. Positive values of FC indicate upregulation, and negative values indicate downregulation. High values emphasized in bold.

**Table 2:** Scores in sensory evaluation of green teas based on different treatments in spring and early and late summer.

Sensory Indexes (SIs) *	Spring (20 April)			Early Summer (6 June)			Late Summer (11 August)		
(SI%)	S1	S2	S3	S1	S2	S3	S1	S2	S3
Appearance (25%)	81.67 ± 0.58 b	86.33 ± 0.58 a	87.67 ± 0.58 a	90.33 ± 0.58 a	88.33 ± 0.58 b	88.33 ± 0.58 b	86.33 ± 0.58 b	88.00 ± 1.00 b	92.00 ± 0.00 a
Aroma (25%)	88.00 ± 0.00 b	85.33 ± 0.58 c	90.33 ± 0.58 a	90.33 ± 0.58 a	87.33 ± 0.58 b	81.67 ± 0.58 c	83.00 ± 0.00 c	88.67 ± 0.58 b	90.33 ± 0.58 a
Infusion color (10%)	85.33 ± 0.58 b	88.33 ± 0.58 a	88.33 ± 0.58 a	90.67 ± 0.58 a	87.67 ± 0.58 b	80.00 ± 0.00 c	87.33 ± 0.58 c	88.67 ± 0.58 b	90.00 ± 0.00 a
Infusion taste (30%)	81.67 ± 0.58 c	85.67 ± 0.58 b	89.00 ± 0.00 a	90.33 ± 0.58 a	87.00 ± 0.00 b	82.00 ± 0.00 c	83.00 ± 1.00 c	86.00 ± 0.00 b	90.00 ± 0.00 a
Infused leaf (color/shape) (10%)	82.67 ± 0.58 c	86.00 ± 0.00 b	88.00 ± 0.00 a	90.00 ± 0.00 a	87.67 ± 0.58 b	88.33 ± 0.58 b	77.67 ± 0.58 c	83.00 ± 1.00 b	88.33 ± 0.58 a
Overall	83.72	86.05	88.83	90.33	87.55	83.93	83.73	87.13	90.42

* Total score of Sensory Indexes (SI) is 100, which includes appearance, aroma, infusion color, infusion taste and infused leaf color/shape contributing 25%, 25%, 10%, 30% and 10% of SI, respectively; a, b and c indicate significant differences between the means obtained based on a Tukey test (*p* < 0.05); tea shoot samples S1, S2 and S3 were collected from tea plants treated without fertilizer and with urea and cow manure, respectively.

## Data Availability

The data is provided in the Supplementary Materials.

## Supplementary Materials

The following are available online, Figure S1: PCA loading plots of metabolites in comparison of S1, S2 and S3, Figure S2: Metabolite–metabolite correlation analysis for tea shoots under three different treatments, Table S1: Extracted and identified compounds from tea shoots under three different treatments, Table S2: The same or different metabolites in tea shoots under three different treatments.

## Sample Availability

Samples of the compounds are not available from the authors.

## Abbreviations

AMIDS, automated mass spectral deconvolution and identification system; ANOVA, one-way analysis of variance; FC, fold change; FDR, false discovery rate; GC–MS, gas chromatography–mass spectrometry; MetPA, metabolomics pathway analysis; NIST, National Institute of Standards and Technology; OPLS-DA, orthogonal-partial least squares-discriminant analysis; PC1, the first principal component; PC2, the second principal component; PCA, principal component analysis; R2X-1, the degree of explanation on the first principal component axis; R2X-2, the degree of explanation on the second principal component axis; t-1, first principal component axis; t-2, second principal component axis; vs., versus.
