# Supplementary material for: Correlation among Metabolic Changes in Tea Plant Camellia sinensis (L.) Shoots, Green Tea Quality and the Application of Cow Manure to Tea Plantation Soils
Source: Molecules. 2021 Oct 13;26(20):6180. doi: 10.3390/molecules26206180 (PMC8538533; doi:10.3390/molecules26206180)
Supplement: Supplementary file 1 [file molecules-26-06180-s001.zip › Additional file Fig. S2.pdf]

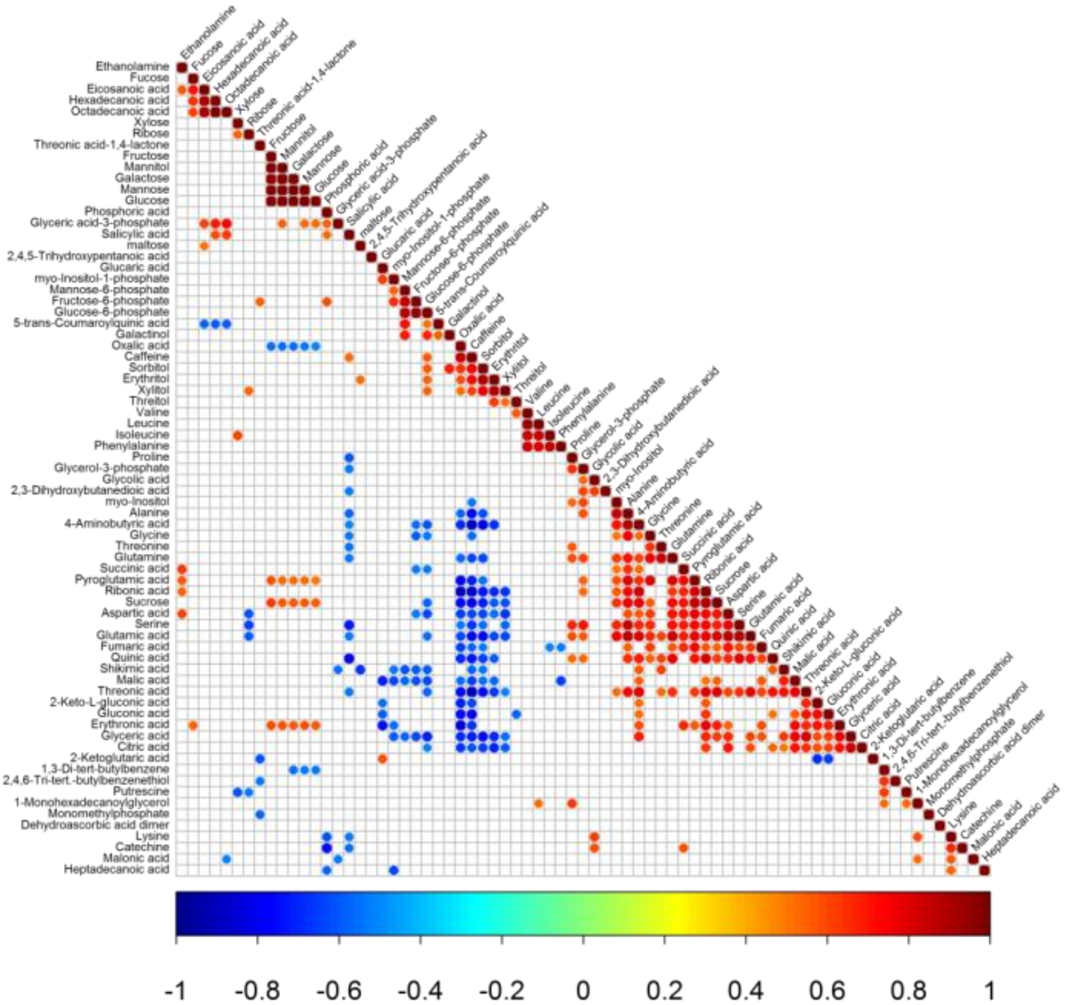

Figure S1. Correlation analysis of metabolite-metabolite. Positive correlation was shown in red and negative correlation was shown in blue.
