# Supplementary figures and images for: Correlation among Metabolic Changes in Tea Plant Camellia sinensis (L.) Shoots, Green Tea Quality and the Application of Cow Manure to Tea Plantation Soils
Source: Molecules. 2021 Oct 13;26(20):6180. doi: 10.3390/molecules26206180 (PMC8538533; doi:10.3390/molecules26206180)

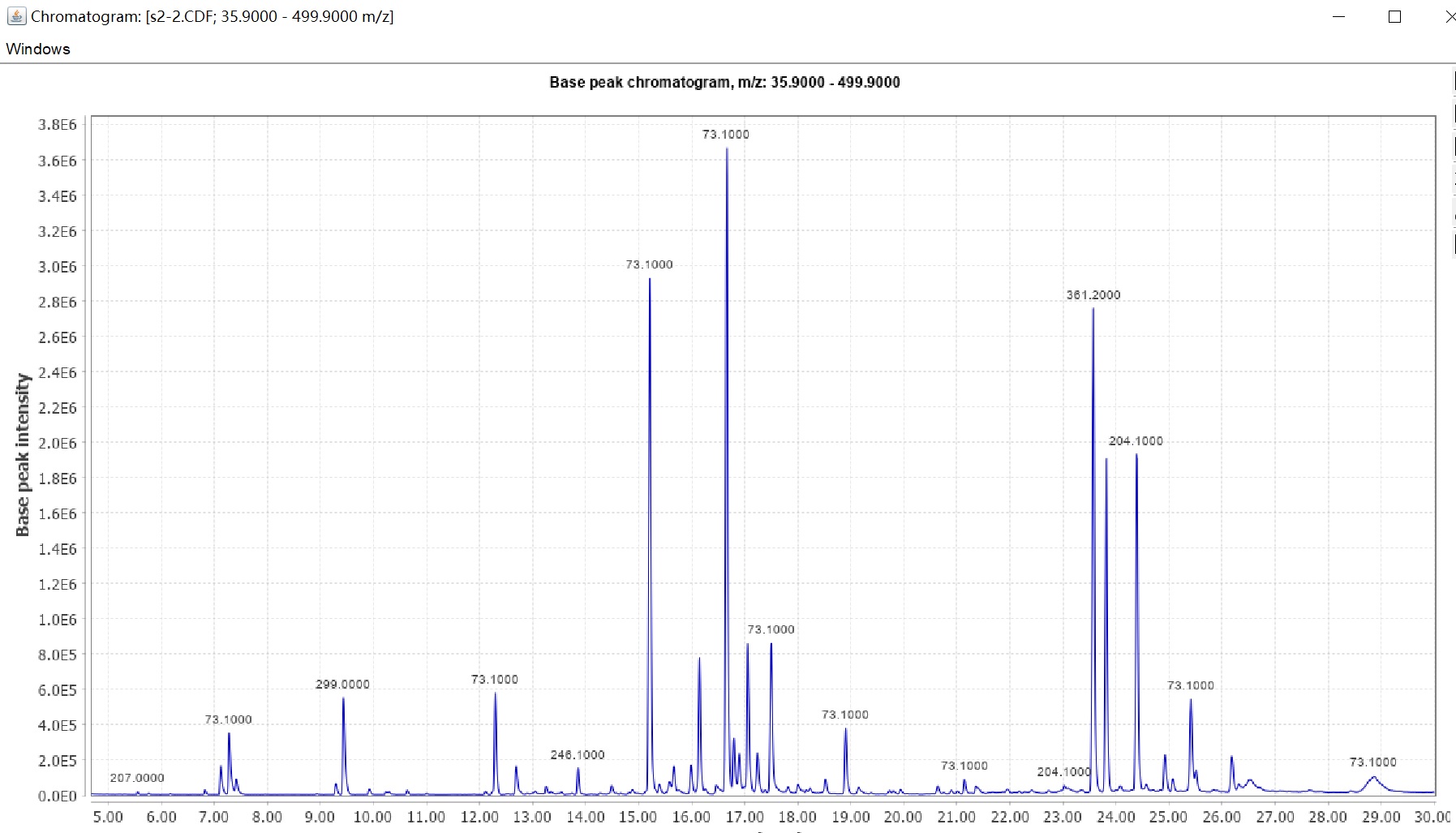

Supplement: Supplementary file 1 [file molecules-26-06180-s001.zip › Evidence/Evidence figure 2 S2-2.jpg]

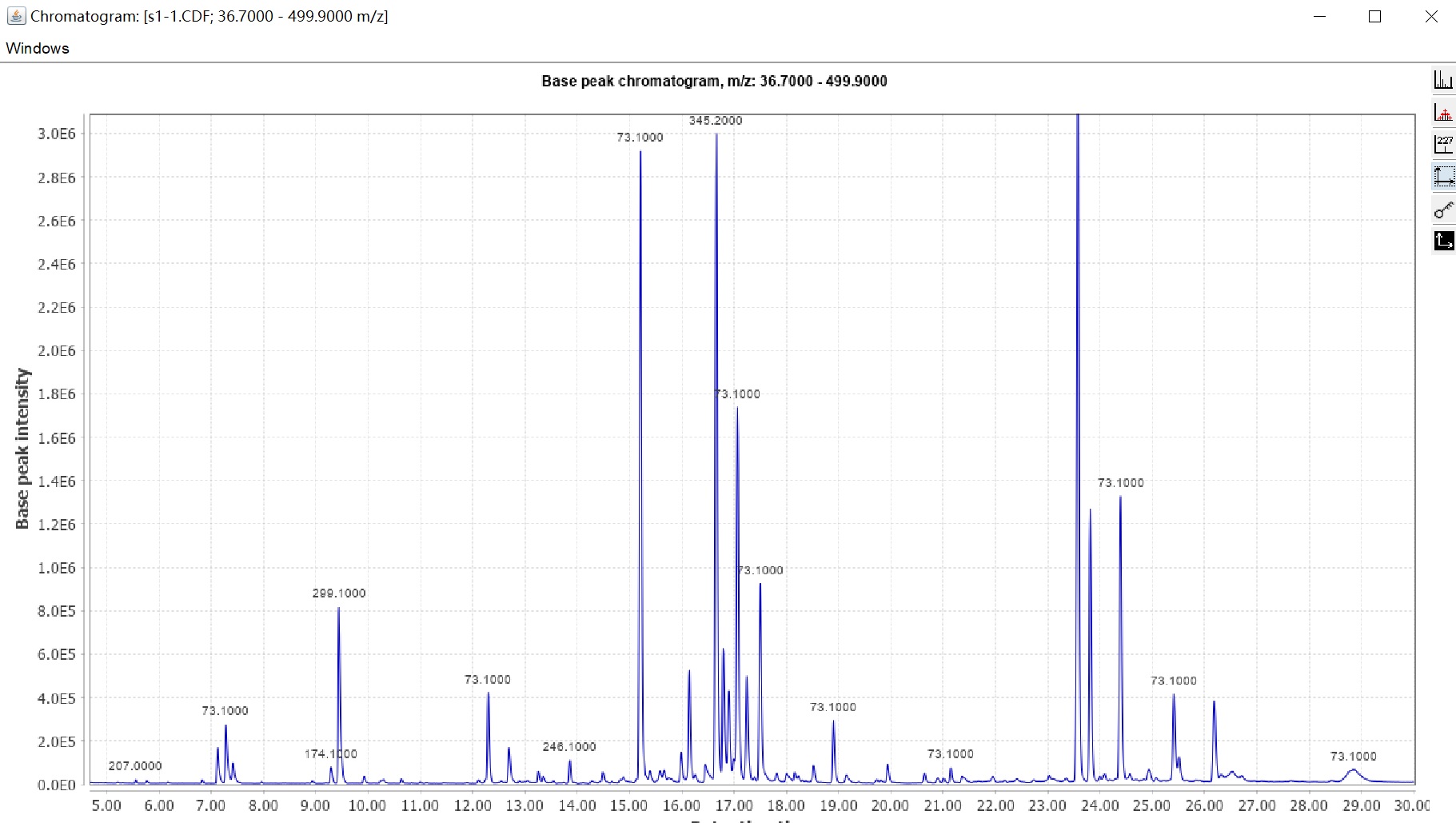

Supplement: Supplementary file 1 [file molecules-26-06180-s001.zip › Evidence/Evidence figure 1 S1-1.jpg]

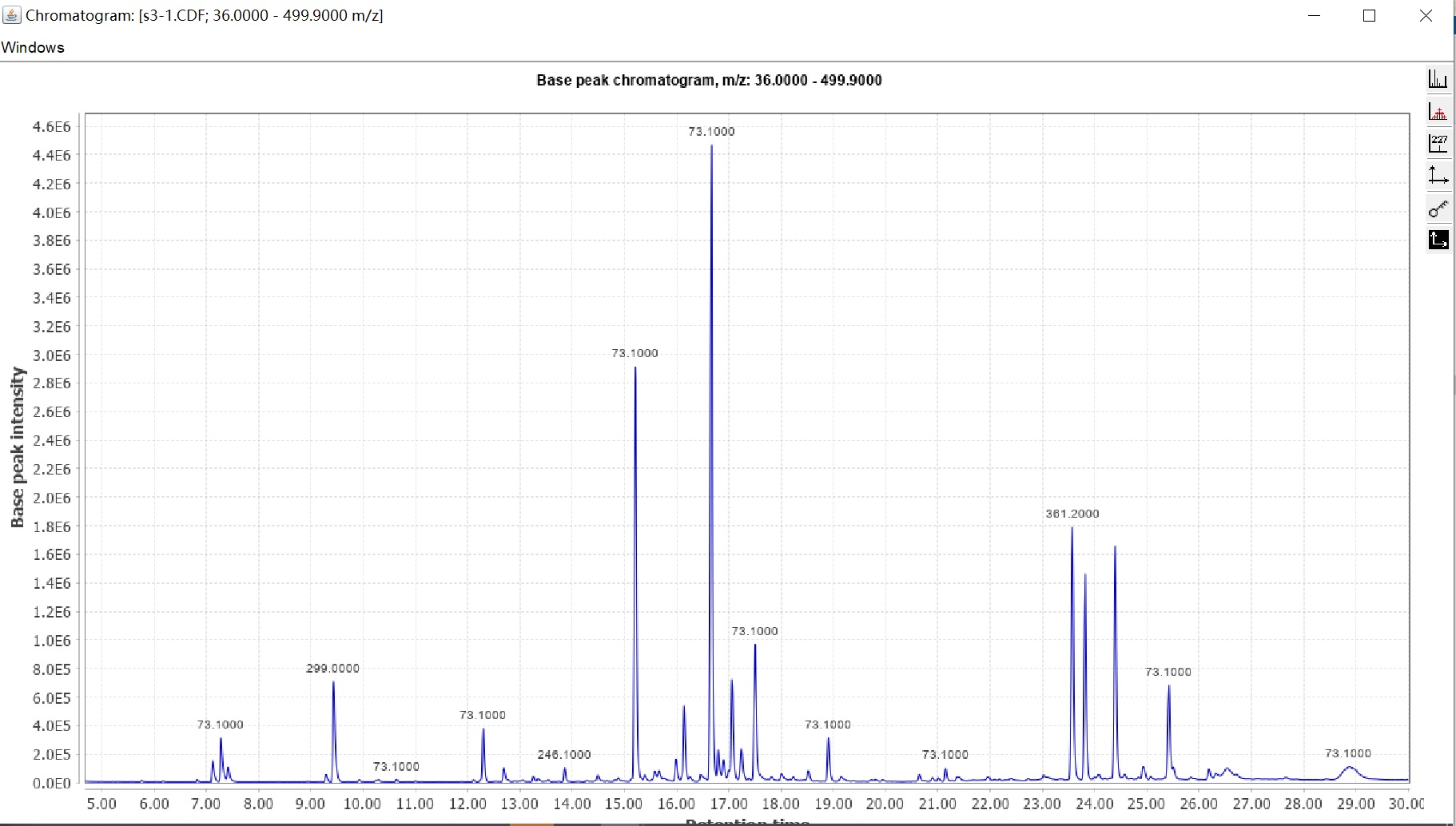

Supplement: Supplementary file 1 [file molecules-26-06180-s001.zip › Evidence/Evidence figure 3 S3-1.jpg]

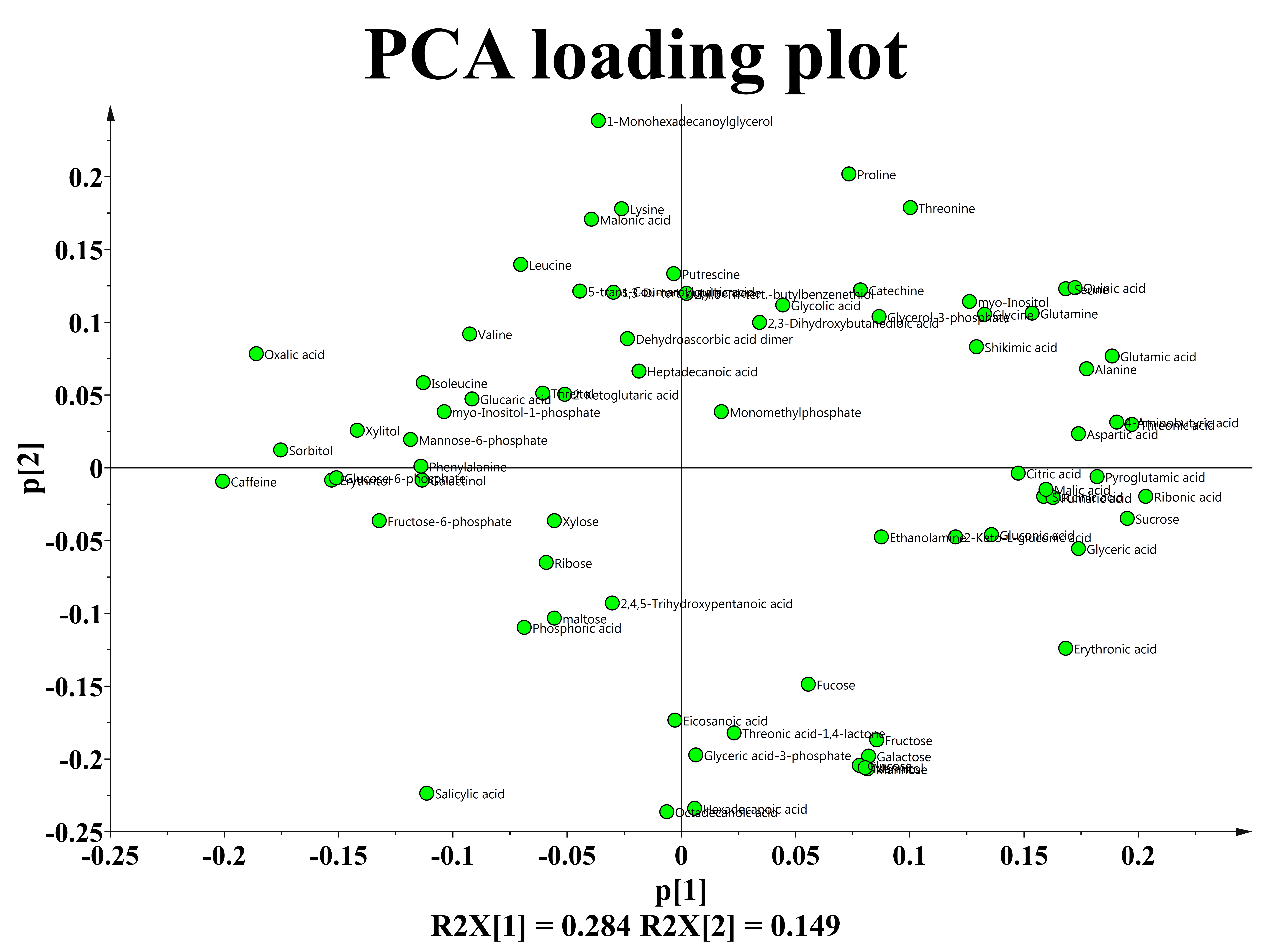

Supplement: Supplementary file 1 [file molecules-26-06180-s001.zip › Fig.S1.tif]
